# Clinical impact of a commercial multiplex pathogen panel for the detection of bacteria in sputum specimens from non-ICU patients with suspected lower respiratory tract infection

**DOI:** 10.1017/ash.2026.10793

**Published:** 2026-07-27

**Authors:** Madeline T. Gemoules, Tristan T. Timbrook, Elizabeth Neuner, Valerie Yuenger, Han Li, Sena Sayood, Tamara Krekel

**Affiliations:** 1 Barnes-Jewish Hospital, USA; 2 Washington University in St Louis, USA

## Abstract

**Objective:**

To evaluate the clinical impact of the BioFire® FilmArray® Pneumonia Panel (BFPP) in sputum specimens from non-intensive care unit (ICU) patients with suspected lower respiratory tract infection.

**Design:**

Retrospective cohort study.

**Setting:**

1400-bed tertiary referral academic medical center.

**Methods:**

Patients who had a sputum specimen with BFPP and standard of care (SOC) culture between 9/1/2022 and 8/31/2024 while on a non-ICU floor were included. An antibiotic change evaluation of actual versus theoretically possible therapy changes was completed at two time points, after BFPP results and after SOC results, overall and by various characteristics, in addition to an assessment of the appropriateness of antibiotic regimens.

**Results:**

189 specimens were included. 48.7% of theoretical changes were acted upon based on BFPP results compared to 87.8% of theoretical changes acted upon based on SOC results. Actual compared to theoretical de-escalation of gram-positive agents occurred more frequently compared to gram-negative agents post-BFPP (43.1% vs 32%) and post-SOC (60.5% vs 38.7%). De-escalation was more frequent in non-neutropenic patients compared to neutropenic patients (39.5% vs 26.1% post-BFPP; 54.7% vs 21.1% post-SOC) and in medicine floor patients compared to oncology floor patients (52.5% vs 23.8% post-BFPP; 72.5% vs 30.2% post-SOC). Appropriateness of antibiotic regimens was higher post-SOC results (77.8%) compared to post-BFPP results (66.1%).

**Conclusions:**

Higher rates of theoretical antibiotic changes were acted upon post-SOC results compared to post-BFPP results, suggesting a disconnect between BFPP results and provider action. Further research is needed to assess factors associated with actionability of the BFPP.

## Introduction

Lower respiratory tract infections (LRTIs) remain a significant contributor to morbidity and mortality.^1^ Traditional approaches for pathogen detection and susceptibility reporting rely on culture-based methods, often resulting in the use of empiric broad-spectrum antibiotics based on patient risk factors while awaiting culture results.^2,3^ The use of rapid diagnostic testing (RDT) may improve and accelerate pathogen detection, leading to antimicrobial optimization and reduced broad-spectrum antibiotics.^4^

Current guidelines by the Infectious Diseases Society of America (IDSA) and the American Thoracic Society for community-acquired, hospital-acquired, and ventilator-associated pneumonia (CAP, HAP, and VAP) offer limited guidance for utilization and interpretation of RDT. Existing recommendations are limited to use of nasal methicillin-resistant *Staphylococcus aureus* (MRSA) cultures/polymerase chain reaction (PCR) to guide decisions on anti-MRSA therapy.^2,3,5^ A IDSA CAP pathway supports molecular testing for bacteria to facilitate the use of directed therapy or antibiotic discontinuation.^6^ Despite these recommendations, evidence to support the clinical utility of multiplex PCR panels remains limited.

The BioFire® FilmArray® Pneumonia Panel (BFPP) is a multiplex PCR panel that provides rapid detection of 33 respiratory targets, including 18 bacteria, eight viruses, and seven antimicrobial resistance genes. BFPP literature has mainly focused on critically ill patients and samples collected from endotracheal aspirate (ETA), bronchoalveolar lavage (BAL), and bronchial washing specimens.^7^ The aim of this study was to explore the usage of the BFPP in non-ICU patients to understand its potential utility in antimicrobial stewardship practices.

## Methods

### Study design and population

This retrospective, single-center study included adult patients admitted to Barnes-Jewish Hospital, a 1,400-bed academic medical center, between 9/1/2022 and 8/31/2024. Patients selected had collection of a sputum specimen with BFPP and aerobic Gram stain and culture while on a non-ICU floor or while in the emergency department if admitted to a non-ICU floor. Clinical diagnosis of LRTI by the care team was assumed given ordering of the BFPP and the challenges in applying criteria for LRTI.^8,9^ No restrictions for BFPP ordering were in place during the study time frame and ordering was at the discretion of the care team. Patients were excluded if they had a concomitant non-LRTI bacterial infection, expired within three days following specimen collection, had a tracheostomy present at the time of specimen collection, or had a contaminated/rejected specimen. Only first non-contaminated/rejected specimens per admission were included. All specimens were fresh clinical specimens collected during routine clinical care and processed in accordance with the panel’s instructions for use.^10^ This study was approved by the Washington University in St. Louis Institutional Review Board with a waiver of informed consent.

### BFPP, sputum specimens, standard of care culture, and study definitions

A description of our microbiology lab’s procedures and definitions used for this data set have been previously published.^11^ Table S2 shows specifics regarding BFPP guidance comments during the study period.

### Study outcomes

An antibiotic change evaluation assessing actual versus theoretically possible therapy changes was completed overall per collected specimen, for each antibiotic for patients on empiric antibiotic(s), and for patients not initially started on empiric antibiotics at two points: (1) after BFPP results, (2) after standard of care (SOC) results. An additional de-escalation evaluation was completed by antibiotic agent spectrum, BFPP semi-quantitative level, time of antibiotic exposure prior to specimen collection, BFPP result time of day, neutrophil count, hospital unit, and BFPP guidance comment. Inappropriate MRSA and inappropriate *Pseudomonas aeruginosa* coverage was reviewed at 24, 48, and 72 hours post-BFPP result. An assessment of the appropriateness of antibiotic regimens and evaluation based on antibiotic spectrum scores was also completed.

### Antibiotic evaluation

Antibiotics evaluated for theoretical and actual changes included gram-positive agents (ceftaroline, linezolid, and vancomycin) and gram-negative agents (ampicillin/sulbactam, cefazolin, cefepime, cefiderocol, ceftazidime, ceftazidime/avibactam, ceftriaxone, imipenem, imipenem/cilastatin/relebactam, levofloxacin, meropenem, moxifloxacin, meropenem/vaborbactam, and piperacillin/tazobactam). Antibiotics not evaluated for changes included aminoglycosides, macrolides, metronidazole, tetracyclines, those used for prophylactic indications, and inhaled antibiotics. Antibiotic de-escalation was defined as a narrowing of antibiotic spectrum or antibiotic discontinuation, and antibiotic escalation was defined as a broadening of spectrum or the addition of an antibiotic intended for LRTI treatment. Each antibiotic was individually assessed for changes.

An assessment of antibiotic appropriateness was completed independently by an infectious diseases pharmacist and a hospital medicine physician at two points: (1) post-BFPP results, (2) post-SOC results. Disagreements in appropriateness were resolved by an infectious diseases physician. Due to the assumption of a clinical diagnosis of LRTI, the guidance in Table S1 was utilized for appropriateness of antibiotic coverage and actual and theoretical change assessment.

Antibiotic spectrum scores were used to evaluate the overall spectrum change per patient between the time of sputum collection and post-BFPP result.^12^ A modified spectrum score was used to assign scores for newer antibiotic agents not available at the time of the system development.^13^

### Data and statistical analysis

Patient and encounter characteristics were summarized using number (percentage) and median (interquartile range [IQR]). For continuous variables, paired t-test or Wilcoxon signed-rank was used and McNemar for binary data given comparisons were made before and after various time points within the same patients, making the data dependently correlated. Using a conditional logistic regression, we performed an exploratory analysis of patient and encounter characteristics associated with any change in antibiotic spectrum score following BFPP result among all patients. This change was measured between the therapy score at time of specimen collection and post-BFPP result. We performed an additional exploratory analysis with a multinomial logistic regression to evaluate factors associated with escalations and de-escalations versus no therapy change among patients started on initial empiric therapy as patients without initial empiric therapy were ineligible for de-escalations. For both models candidate variables included age ≥65 years, postguidance period, sex, race, Charlson Comorbidity Index >3, neutropenia, infection criteria, leukopenia, leukocytosis, fever, hypothermia, severe immunocompromise, respiratory support, suspected LRTI type, prior antibiotic exposure, duration of antibiotic exposure before BFPP collection, time to BFPP collection, active COVID-19 infection, and baseline antibiotic spectrum score. Variables were first assessed in univariable multinomial logistic regression models, and those with *P* < .15 were considered for inclusion in the final multivariable multinomial model. All analyses were performed in IBM® SPSS® Statistics version 25 and R version 4.4.1. Results were reported in concordance with STrengthening the Reporting of Observational Studies in Epidemiology (STROBE) guidelines.^14^

## Results

### Patient population

A total of 784 sputum specimens were screened; 189 specimens from 187 unique patients met inclusion criteria (Figure S1). The mean age of included patients was 60.1 years, most patients were male (57.8%) and White (63%), and the median Charlson comorbidity index was 3 (IQR 2–6). Twenty-eight percent of patients had chronic obstructive pulmonary disease, 10% asthma, 31.7% diabetes mellitus, 16.4% hematologic malignancy, and 11.1% neutropenia at the time of specimen collection. Few patients had a history of a multidrug-resistant organism within six months prior to specimen collection, with the most common being *P. aeruginosa* (5.3%). Fifty percent of patients had a white blood cell count >10,000 cells/mcL and 16% had a temperature ≥ 38°C within 24 hours of specimen collection. Most LRTIs occurred in patients admitted for <48 hours prior to specimen collection and classified as community-acquired (66.1%). Most specimens were expectorated sputum (75.1%) and were obtained while patients were admitted to a medicine (58.2%) or oncology (32.8%) unit. One-hundred and forty-four specimens (76.2%) were collected after antibiotic exposure with a median number of antibiotics of 1 (IQR 1–2) and a median duration of antibiotic exposure of 21.7 (IQR 8.7–38.2) hours prior to collection. The most administered antibiotics prior to collection were cefepime (36%), vancomycin (31.2%), and azithromycin (25.4%) (Table 1). Table S3 shows the distribution of BFPP bacterial target detections.

**Table 1.** Patient & antibiotic characteristics Table 1 long description.

Patient characteristic	N = 189^1^
Age, years, mean (SD)	60.1 (14.6)
Male, n (%)	109 (57.8)
Race, n (%)	
White	119 (63)
Black	61 (32.3)
Other	6 (3.2)
Unable to answer/declined	3 (1.6)
Season of testing, n (%)	
Winter (December–February)	44 (23.3)
Spring (March–May)	67 (35.4)
Summer (June–August)	43 (22.8)
Fall (September–November)	35 (18.5)
Charlson comorbidity index, median (IQR)	3 (2–6)
Select comorbidities, n (%)	
Asthma	19 (10)
Chronic obstructive pulmonary disease	53 (28)
Diabetes mellitus	60 (31.7)
Immunocompromising condition, n (%)^2^	
Solid organ transplant	6 (3.2)
Hematologic malignancy	31 (16.4)
Hematopoietic cell transplant	11 (5.8)
People with HIV	8 (4.2)
Neutropenia (ANC < 1,000 cells/mcL)	21 (11.1)
History of MDR infection/colonization within 6 months of sputum collection, n (%)	
Extended-spectrum beta-lactamase Enterobacterales	1 (0.5)
Carbapenem-resistant Enterobacterales	0 (0)
Carbapenem-resistant *Acinetobacter calcoaceticus-baumannii* complex	0 (0)
*Pseudomonas aeruginosa*	10 (5.3)
Methicillin-resistant *Staphylococcus aureus*	2 (1.1)
Infectious signs/symptoms within 24 hours of sputum collection, n/N (%)	
WBC > 10,000 cells/mcL	90/180 (50)
WBC < 4,000 cells/mcL	33/180 (18.3)
Temperature ≥ 38°C	30/188 (16)
Temperature ≤ 36°C	7/188 (3.7)
Location at sputum collection, n (%)	
Emergency department	8 (4.2)
Floor	181 (95.8)
Hospital unit, n (%)	
Cardiology	6 (3.2)
Medicine	110 (58.2)
Oncology	62 (32.8)
Surgery	7 (3.7)
Other^3^	4 (2.1)
Hospital LOS prior to sputum order, days, median (IQR)	1.1 (0.3–3.0)
Hospital LOS prior to sputum collection, days, median (IQR)	1.5 (0.6–3.5)
Respiratory support at time of sputum collection, n (%)	
None	95 (50.3)
Nasal cannula	94 (49.7)
Specimen type, n (%)	
Expectorated sputum	142 (75.1)
Induced sputum	47 (24.9)
BFPP result time of day, n (%)	
0700–1,459	70 (37)
1,500–2,259	73 (38.6)
2,300–0659	46 (24.4)
BFPP result guidance comment, n (%)	
Prechange^4^	100 (52.9)
Postchange^5^	89 (47.1)
Suspected infection type, n (%)	
Community-acquired LRTI	125 (66.1)
Hospital-acquired LRTI	64 (33.9)
Active COVID-19 infection, n (%)	8 (4.2)
Infectious diseases consult during hospitalization, n (%)	51 (27)
**Antibiotic characteristic**	**N = 189**
Antibiotic exposure prior to sputum collection, n (%)	144 (76.2)
Duration of antibiotic exposure prior to sputum collection, hours, median (IQR)	21.7 (8.7–38.2)
Antibiotics at sputum collection, n (%)^6^	
Ampicillin/sulbactam	6 (3.2)
Azithromycin	48 (25.4)
Cefepime	68 (36)
Ceftriaxone	35 (18.5)
Levofloxacin	5 (2.6)
Linezolid	9 (4.8)
Meropenem	13 (6.9)
Piperacillin/tazobactam	10 (5.3)
Vancomycin	59 (31.2)
Other^7^	11 (5.8)
Number of antibiotics at sputum collection, median (IQR)	1 (1–2)
LOS following BFPP result, days, median (IQR)	4.1 (2.1–7.4)

ANC, absolute neutrophil count; BFPP, BioFire® FilmArray® Pneumonia Panel; HIV, human immunodeficiency virus; IQR, interquartile range; LOS, length of stay; LRTI, lower respiratory tract infection; MDR, multidrug-resistant; SD, standard deviation.

1 A total of 189 sputum specimens from 187 patients; 2 patients were included two times due to meeting criteria during two distinct admissions.

2 Immunocompromising conditions were not considered mutually exclusive.

3 Other hospital units: neurology (n = 2), obstetrics (n = 1), urology (n = 1).

4 Prechange guidance comment: Rapid molecular analysis has NOT detected bacterial targets (for a list of targets evaluated, refer to the interpretive data for this specimen). Correlation of molecular analysis with culture results is recommended.

5 Postchange guidance comment: No bacterial targets detected by molecular analysis. A negative molecular panel strongly supports discontinuation of anti-MRSA and anti-pseudomonal therapy for the treatment of pneumonia due to MRSA and *P. aeruginosa* (refer to interpretive data for full list of targets evaluated). Correlation of molecular analysis with culture results is recommended.

6 Percentages add up to greater than 100 due to patients being on multiple antibiotics concurrently.

7 Other antibiotics: amoxicillin/clavulanate (n = 2), aztreonam (n = 1), cefiderocol (n = 1), ceftriaxone (n = 1), doxycycline (n = 3), minocycline (n = 2), and moxifloxacin (n = 1).

### Antibiotic change evaluation (Table 2)

Based on the results of the BFPP, a theoretical change was possible in 63% (n = 119/189) of specimens, with an actual change occurring in 30.7% (n = 58/189) of specimens, demonstrating 48.7% of theoretical changes were acted upon. SOC results established a theoretical change in 25.9% (n = 49/189) of specimens, with an actual change being made in 22.8% (n = 43/189) of specimens, demonstrating 87.8% of theoretical changes were acted upon. BFPP results supported a theoretical change in 65.5% (n = 152/232) of antibiotics, with an actual change occurring in 26.3% (n = 61/232) of antibiotics, demonstrating 40.1% of theoretical changes were acted upon. SOC results established a theoretical change in 50.2% (n = 106/211) of antibiotics, with an actual change being made in 25.1% (n = 53/211) of antibiotics, demonstrating 50% of theoretical changes were acted upon. Most theoretical and actual changes were de-escalations for both BFPP and SOC results.

**Table 2.** Antibiotic change evaluation Table 2 long description.

	Based on BFPP			Based on SOC		
	Theoretical change	Actual change	Actual compared to theoretical change	Theoretical change	Actual change	Actual compared to theoretical change
**Any change per specimen, n/N (%)**	119/189 (63)	58/189 (30.7)	58/119 (48.7)	49/189 (25.9)	43/189 (22.8)	43/49 (87.8)
**>1 change per specimen, n/N (%)**	53/189 (28)	17/189 (9)	17/53 (32.1)	30/189 (15.9)	12/189 (6.3)	12/30 (40)
**Number of antibiotic changes, median (IQR)**	1 (0–2)	0 (0–1)	–	0 (0–1)	0 (0–0)	–
**Changes by antibiotic, n/N (%)**	152/232 (65.5)	61/232 (26.3)	61/152 (40.1)	106/211 (50.2)	53/211 (25.1)	53/106 (50)
Escalation	5/152 (3.3)	6/152 (3.9)	6/5 (>100)^1^	1/106 (0.9)	2/106 (1.8)	2/1 (>100)^2^
De-escalation	147/152 (96.7)	55/152 (36.2)	55/147 (37.4)	105/106 (99.1)	51/106 (48.1)	51/105 (48.6)
**Changes for those not on antibiotics, n/N (%)**						
Continue no antibiotics	18/35 (51.4)	23/35 (65.7)	23/18 (>100)^3^	26/31 (83.9)	29/31 (93.5)	29/26 (>100)^4^
Escalation	17/35 (48.6)	12/35 (34.3)	12/17 (70.6)	5/31 (16.1)	2/31 (6.5)	2/5 (40)

BFPP, BioFire® FilmArray® Pneumonia Panel; SOC, standard of care.

1 More actual escalations occurring compared to theoretical escalations based on BFPP results.

2 More actual escalations occurring compared to theoretical escalations based on SOC results.

3 More actual continuation of no antibiotics compared to theoretical continuation of no antibiotics based on BFPP results.

4 More actual continuation of no antibiotics compared to theoretical continuation of no antibiotics based on SOC results.

Thirty-five patients did not have empiric antibiotics initiated at the time of specimen collection. BFPP results supported a theoretical escalation in 48.6% (n = 17/35) of patients, with actual escalation occurring in 34.3% (n = 12/35) of patients, demonstrating 70.6% of theoretical escalations were acted upon, and SOC results established a theoretical escalation in 16.1% (n = 5/31) of patients, with actual escalation occurring in 6.5% (n = 2/31) of patients, demonstrating 40% of theoretical escalations were acted upon.

### De-escalation evaluation (Table 3)

Of the 232 antibiotics included in the BFPP analysis, 63.4% (n = 147/232) were subject to theoretical de-escalation, with an actual de-escalation occurring in 23.7% (n = 55/232), demonstrating 37.4% of theoretical de-escalations were acted upon. SOC results established a theoretical de-escalation in 49.8% (n = 105/211) of antibiotics, with an actual de-escalation occurring in 24.2% (n = 51/211), demonstrating 48.6% of theoretical de-escalations were acted upon. When evaluated by antibiotic agent spectrum, 49% and 51% of theoretical de-escalations based on BFPP results and 41% and 59% of theoretical de-escalations based on SOC results were for gram-positive and gram-negative agents, respectively. Actual compared to theoretical de-escalation of gram-positive agents after BFPP results occurred more frequently (43.1%, n = 31/72) compared to de-escalation for gram-negative agents (32%, n = 24/75). A similar de-escalation trend was seen in response to SOC results (60.5%, n = 26/43 vs 38.7%, n = 24/62).

**Table 3.** De-escalation evaluation Table 3 long description.

	Based on BFPP			Based on SOC		
	Theoretical de-escalation	Actual de-escalation	Actual compared to theoreticalde-escalation	Theoretical de-escalation	Actual de-escalation	Actual compared to theoretical de-escalation
**Overall by antibiotic, n/N (%)**	147/232 (63.4)	55/232 (23.7)	55/147 (37.4)	105/211 (49.8)	51/211 (24.2)	51/105 (48.6)
**By antibiotic agent spectrum, n/N (%)**						
Gram-positive	72/147 (49)	31/147 (21.1)	31/72 (43.1)	43/105 (41)	26/105 (24.8)	26/43 (60.5)
Gram-negative	75/147 (51)	24/147 (16.3)	24/75 (32)	62/105 (59)	24/105 (22.9)	24/62 (38.7)
**By BFPP semi-quantitative level, n/N (%)**						
Low (10^4^–10^5^ copies/mL)	43/147 (29.3)	20/147 (13.6)	20/43 (46.5)	29/105 (27.6)	19/105 (18.1)	19/29 (65.5)
High (10^6^–10^7^ copies/mL)	41/147 (27.9)	16/147 (10.9)	16/41 (39)	35/105 (33.3)	24/105 (22.9)	24/35 (68.6)
**By antibiotic exposure, n/N (%)**						
>24 hours	12/147 (8.2)	4/147 (2.7)	4/12 (33.3)	12/105 (11.4)	7/105 (6.7)	7/12 (58.3)
≤ 24 hours	71/147 (48.3)	30/147 (20.4)	30/71 (42.3)	50/105 (47.6)	36/105 (34.3)	36/50 (72)
**By BFPP result time of day, n/N (%)**						
0700–1,459	53/147 (36)	20/147 (13.6)	20/53 (37.7)	39/105 (37.1)	15/105 (14.3)	15/39 (38.5)
1,500–2,259	57/147 (38.8)	22/147 (15)	22/57 (38.6)	39/105 (37.1)	16/105 (15.2)	16/39 (41)
2,300–0659	37/147 (25.2)	13/147 (8.8)	13/37 (35.1)	27/105 (25.8)	20/105 (19)	20/27 (74.1)
**By neutrophil count, n/N (%)**						
ANC<1,000 cells/mcL	23/147 (15.6)	6/147 (4.1)	6/23 (26.1)	19/105 (18.1)	4/105 (3.8)	4/19 (21.1)
ANC ≥ 1,000 cells/mcL	124/147 (84.4)	49/147 (33.3)	49/124 (39.5)	86/105 (81.9)	47/105 (44.8)	47/86 (54.7)
**By hospital unit, n/N (%)**						
Oncology	84/147 (55.1)	20/147 (13.6)	20/84 (23.8)	63/105 (60)	19/105 (18.1)	19/63 (30.2)
Medicine	61/147 (41.5)	32/147 (21.7)	32/61 (52.5)	40/105 (38.2)	29/105 (27.6)	29/40 (72.5)
**By BFPP guidance comment, n/N (%)**						
Negative BFPP prechange^1^	35/50 (70)	15/50 (30)	15/35 (42.9)	–^3^	–^3^	–^3^
Negative BFPP postchange^2^	17/40 (42.5)	9/40 (22.5)	9/17 (52.9)	–^3^	–^3^	–^3^

ANC, absolute neutrophil count; BFPP, BioFire® FilmArray® Pneumonia Panel; SOC, standard of care.

1 Prechange guidance comment: Rapid molecular analysis has NOT detected bacterial targets (for a list of targets evaluated, refer to the interpretive data for this specimen). Correlation of molecular analysis with culture results is recommended.

2 Postchange guidance comment: No bacterial targets detected by molecular analysis. A negative molecular panel strongly supports discontinuation of anti-MRSA and anti-pseudomonal therapy for the treatment of pneumonia due to MRSA and *P. aeruginosa* (refer to interpretive data for full list of targets evaluated). Correlation of molecular analysis with culture results is recommended.

3 No guidance comment is associated with SOC results.

Theoretical antibiotic de-escalations by BFPP semi-quantitative level were similar for high (10^6^–10^7^ copies/mL) semi-quantitative levels (27.9%, n = 41/147) and low (10^4^–10^5^ copies/mL) semi-quantitative levels (29.3%, n = 43/147), with similar rates of actual de-escalations (10.9%, n = 16/147 vs 13.6%, n = 20/147), and actual compared to theoretical de-escalations (39%, n = 16/41 vs 46.5%, n = 20/43). More theoretical de-escalations based on BFPP results were possible for patients who had antibiotics started <24 hours before time of specimen collection compared to those with >24 hours of antibiotics (48.3%, n = 71/147 vs 8.2%, n = 12/147), with an actual de-escalation occurring 20.4% (n = 30/147) versus 2.7% (n = 4/147) of the time, demonstrating 42.3% and 33.3% of theoretical changes were acted upon, respectively. This trend continued when looking at response to SOC results, with 72% and 58.3% of theoretical changes being acted upon, respectively. When analyzed by BFPP resulting time of day, similar rates of theoretical changes were acted upon. Compared to non-neutropenic and medicine floor patients, respectively, neutropenic patients (26.1%, n = 6/23 vs 39.5%, n = 49/124) and oncology floor patients (23.8%, n = 20/84 vs 52.5%, n = 32/61) had lower rates of theoretical changes based on BFPP acted upon, which was also reflected in lower rates of changes based on SOC being acted upon. The guidance comment associated with a negative BFPP changed throughout the study period; a lower number of theoretical changes were acted upon pre versus postcomment change (42.9%, n = 15/35 vs 52.9%, n = 9/17).

### Appropriateness evaluation

Rates of inappropriate *P. aeruginosa* coverage were higher than rates of inappropriate MRSA coverage at 24, 48, and 72 hours from BFPP result (30% vs 21.1%, 20.2% vs 12.2%, 17.2% vs 9.5%, respectively). When assessing appropriateness of antibiotic regimens after BFPP results and after SOC results, there was 8.5% (n = 16/189) and 10.6% (n = 20/189) disagreement, respectively, between the initial reviewers, which were resolved by the third reviewer. Appropriateness of antibiotic regimens after BFPP results was 66.1% (n = 125/189), while appropriateness after SOC results was 77.8% (n = 147/189).

### Spectrum score evaluation

The median spectrum score between specimen collection and BFPP result was the same for all patients but was lower post-BFPP result than at the time of specimen collection for patients on initial antibiotic therapy, primarily driven by azithromycin discontinuation (Table S4; Figure S2). A suspected infection type of community-acquired LRTI compared to hospital-acquired LRTI (adjusted odds ratio [aOR] 3.50; 95% confidence interval [CI] 1.73–7.39; *P* < .01) and every ten-point increase in initial spectrum score (aOR 1.21; 95% CI 1.04–1.43; *P* = .02) were associated with any change in spectrum score (Table S5). In multinomial logistic regression of patients on initial therapy at specimen collection, de-escalation (vs no change) was associated with higher baseline spectrum score (per 10-point increase; aOR 1.85, 95% CI 1.38–2.49, *P* < .001) and age ≥65 years (aOR 2.66, 95% CI 1.21–5.88, *P* = .015).

## Discussion

Previous published literature on the clinical utility of the BFPP primarily focuses on deeper respiratory specimens or combines specimen types for analyses, making the role of the BFPP in solely sputum specimens less clear for antimicrobial stewardship practices.^6,15–22^ This study evaluated the clinical impact of the BFPP in relation to SOC culture from 189 sputum specimens from non-ICU patients with suspected LRTI, which, to our knowledge, is the largest analysis of exclusively sputum specimens in this patient population published to date. As previously published, this cohort resulted in an overall positive percent agreement of 96.3%, negative percent agreement of 54.9%, positive predictive value of 26.3%, and negative predictive value of 98.9%.^11^

In the included patients, BFPP results led to the identification of a theoretical antibiotic change 63% of the time; however, less than half (48.7%) were acted upon, compared to 87.8% of theoretical antibiotic changes based on SOC results being acted upon. This discrepancy suggests a disconnect between BFPP results and provider action on those results. Provider familiarity with interpreting SOC results may have influenced the higher rate of actual antibiotic changes compared to BFPP results, since the BFPP has only been available at our institution since September 2021, and familiarity and related impact of multiplex PCR tests is known to change over time.^22^

Most commonly, the theoretical change(s) based on BFPP results was antibiotic de-escalation, which is not surprising given that 47.6% of specimens resulted as BFPP negative, *Haemophilus influenzae* and *Streptococcus* species were the most common bacterial targets detected, and the high number of patients on empiric cefepime and vancomycin despite the majority having community-acquired LRTI.^11^ However, providers were more reliable in escalating therapy rather than de-escalating therapy based on BFPP results which could be reflective of a lack of understanding of the BFPP’s performance characteristics, particularly the negative predictive value, or of the epidemiology of LRTI, with resistant bacterial infections being overemphasized in empiric regimens.

Factors associated with actionability of infectious diseases RDT have been infrequently explored. In our data, actual de-escalation of gram-positive agents occurred more frequently than gram-negative agents at both timepoints. This was not surprising, given the increased familiarity with utilizing nasal MRSA culture or PCR to de-escalate therapy for respiratory infections.^23^ During the study period, it was not uncommon for providers to order nasal MRSA cultures in addition to the BFPP, likely due to a lack of understanding that this was duplicative. We did not collect data regarding testing or results of nasal MRSA cultures, which is a limitation since negative nasal MRSA culture results may have also influenced de-escalation of gram-positive agents.

No clinically significant differences in the rates of theoretical changes being acted upon were seen when analyzed by BFPP semi-quantitative level or by BFPP result time of day; both possibly due to being underpowered to detect differences, the former possibly due to provider unfamiliarity with semi-quantitative reporting, and the latter likely due to the lack of a dedicated antimicrobial stewardship intervention at our institution related to BFPP results. This is also reflected in the increase in appropriate antibiotic regimens post-SOC by almost 12% compared to post-BFPP.

Ideally, specimen collection would occur prior to antibiotic administration, however, most patients in this study received antibiotics prior to collection. Patients with ≤24 hours of antibiotic exposure prior to sputum collection had higher rates of theoretical changes acted upon compared to patients with over 24 hours of antibiotic exposure both post-BFPP result and post-SOC result, likely reflecting concern of diagnostic/culture sterilization with antibiotic exposure prior to specimen collection. Neutropenic and oncology patients had lower rates of theoretical changes acted upon compared to non-neutropenic and medicine floor patients, respectively, which was primarily driven by the continuation of antibiotics that covered *P. aeruginosa* in the absence of BFPP or SOC results that supported continuation. Current guideline recommendations for neutropenic fever recommend initiation of empiric antibiotics with activity against *P. aeruginosa* but differ in their recommendations on whether this coverage should be de-escalated once a pathogen is identified.^24–26^ Given that published studies assessing de-escalation strategies in neutropenic patients primarily assess fever of unknown origin, the primary practice at our institution is to continue activity against *P. aeruginosa*, which explains the lower rate of actual antibiotic changes in these patients.

The microbiology guidance comment associated with a negative BFPP changed half-way through the study period to more clearly point out that a negative molecular panel strongly supports discontinuation of anti-MRSA and anti-*P. aeruginosa* therapy for the treatment of pneumonia. Similar to previous studies evaluating nudge comments, we saw an increase in de-escalation of anti-MRSA and anti-*P. aeruginosa* therapy postguidance comment change, adding to the literature supporting this relatively simple antimicrobial stewardship intervention.^27,28^ More research is needed on the addition of active antimicrobial stewardship team interventions based on BFPP results.

This was a retrospective, single-center study at an academic medical center. Diagnosis of LRTI was assumed based on BFPP ordering by the treating provider and was not validated by diagnosis code correlation or radiographic review, therefore, was likely ordered in patients with other respiratory diseases, such as chronic obstructive pulmonary disease exacerbation. Utilizing diagnosis codes, clinical symptoms, and/or radiographic findings of LRTI may have improved selection of patients with true LRTI and helped with assessment of regimen appropriateness, however, this study still appropriately evaluates the impact of the BFPP on antimicrobial stewardship. Further studies limited to confirmed LRTI, including the impact of BFPP on clinical outcomes, are an area for future research. The epidemiology of the bacterial targets seen at our institution, empiric antibiotic treatment practices, low incidence of patients with previous resistant organism colonization or infection, and higher patient acuity may limit external validity and should be taken into consideration for other institutions looking to this data.

Overall, higher rates of theoretical antibiotic changes were acted upon post-SOC results compared to post-BFPP results, suggesting a disconnect between BFPP results and provider action. Suggested guideline recommendations on molecular bacterial testing from the IDSA note that such testing should be restricted to patients in whom timely pathogen determination may allow a more directed therapy or discontinuation of unnecessary antibiotics in CAP. Our research supports these recommendations given the high proportion of patients where the BFPP result could allow for more directed therapy, though comparative studies with control groups are needed. Future research is also needed to elucidate factors associated with actionability of RDT such that diagnostic stewardship may drive appropriate testing among patients who are likely to truly benefit.

## Supporting information

10.1017/ash.2026.10793.sm001 Gemoules et al. supplementary material Gemoules et al. supplementary material

## Data Availability

Research data are not shared.

## Supplementary material

The supplementary material for this article can be found at https://doi.org/10.1017/ash.2026.10793.

## Table 1. Long description

The table presents patient and antibiotic characteristics for 189 sputum specimens from 187 unique patients. It includes 23 rows and 10 columns. Column headers are Patient characteristic, N = 189, Age, years, mean (SD), Male, n (%), Race, n (%), Season (May-August), n (%), Charlson comorbidity index, median (IQR), Select comorbidities, n (%), Immunocompromising condition, n (%), People at risk, n (%), History of M D R infection/colonization within 6 months of sputum collection, n (%), Infectious signs/symptoms within 24 hours of sputum collection, n/n (%), Hospital unit, n (%), Hospital LOS prior to sputum order, days, median (IQR), Hospital LOS prior to sputum collection, day, median (IQR), Respiratory support at time of sputum collection, n (%), Specimen type, n (%), B F P P result time of day, n (%), B F P P result, n (%), Suspected infection type, n (%), Hospital-acquired L R T I, n (%), Active C O V I D-19 infection, n (%), Infectious diseases consult during hospitalization, n (%), Antibiotic characteristic, Antibiotic exposure prior to sputum collection, n (%), Duration of antibiotic exposure prior to sputum collection, hours, median (IQR), Antibiotics at sputum collection, n (%), and Number of antibiotics at sputum collection, median (IQR). Row 1: Patient characteristic, N = 189, Age, years, mean (SD), 60.1 (14.6), Male, n (%), 109 (57.8), Race, n (%), White, 119 (63), Black, 61 (32.3), Other, 6 (3.2), Unable to answer/declined, 3 (1.6), Season (May-August), n (%), Winter (December-February), 44 (23.3), Spring (March-May), 62 (32.8), Summer (June-August), 43 (22.8), Fall (September-November), 35 (18.5), Charlson comorbidity index, median (IQR), 3 (2-4), Select comorbidities, n (%), Asthma, 19 (10), Chronic obstructive pulmonary disease, 53 (28), Diabetes mellitus, 60 (31.7), Immunocompromising condition, n (%), Solid organ transplant, 6 (3.2), Hematologic malignancy, 31 (16.4), Hematopoietic cell transplant, 11 (5.8), People at risk, n (%), Neutropenia (A N C < 1,000 cells/mcL), 21 (11.1), History of M D R infection/colonization within 6 months of sputum collection, n (%), Extended-spectrum beta-lactamase Enterobacterales, 1 (0.5), Carbapenem-resistant Enterobacterales, 0 (0), Carbapenem-resistant Acinetobacter calcoaceticus-baumannii complex, 0 (0), Pseudomonas aeruginosa, 10 (5.3), Methicillin-resistant Staphylococcus aureus, 2 (1.1), Infectious signs/symptoms within 24 hours of sputum collection, n/n (%), W B C > 10,000 cells/mcL, 90/180 (50), W B C < 4,000 cells/mcL, 31/180 (18.3), Temperature 38C, 30/180 (16), Hospital unit, n (%), Medicine, 110 (58.2), Oncology, 62 (32.8), Surgery, 10 (5.3), Other, 5 (2.6), Hospital LOS prior to sputum order, days, median (IQR), 1.1 (0.3-3.0), Hospital LOS prior to sputum collection, day, median (IQR), 1.5 (0.6-3.5), Respiratory support at time of sputum collection, n (%), Nasal cannula, 95 (50.3), Specimen type, n (%), Expectorated sputum, 142 (75.1), Induced sputum, 47 (24.9), B F P P result time of day, n (%), 0700-1,459, 70 (37), 1,500-2,259, 73 (38.6), 2,300-0,659, 46 (24.4), B F P P result, n (%), Prechange, 100 (52.9), Postchange, 89 (47.1), Suspected infection type, n (%), Community-acquired, 125 (66.1), Hospital-acquired, 64 (33.9), Active C O V I D-19 infection, n (%), 8 (4.2), Infectious diseases consult during hospitalization, n (%), 51 (27), Antibiotic characteristic, Antibiotic exposure prior to sputum collection, n (%), 144 (76.2), Duration of antibiotic exposure prior to sputum collection, hours, median (IQR), 21.7 (8.7-38.2), Antibiotics at sputum collection, n (%), Ampicillin/sulbactam, 6 (3.2), Azithromycin, 48 (25.4), Cefepime, 68 (36), Ceftriaxone, 35 (18.5), Ceftobiprole, 5 (2.6), Clindamycin, 9 (4.8), Levofloxacin, 11 (5.8), Linezolid, 10 (5.3), Meropenem, 10 (5.3), Piperacillin/tazobactam, 39 (20.7), Vancomycin, 59 (31.2), Other, 11 (5.8), Number of antibiotics at sputum collection, median (IQR), 1 (0-2).

Navigate back to Table 1..

## Table 2. Long description

The table compares antibiotic changes based on BioFire FilmArray Pneumonia Panel (BFPP) and standard of care (SOC). It has 12 rows and 7 columns. Column headers are Theoretical change, Actual change, Actual compared to theoretical change, Theoretical change, Actual change, Actual compared to theoretical change. Row labels include Any change per specimen, n/N (%), >1 change per specimen, n/N (%), Number of antibiotic changes, median (IQR), Changes by antibiotic, n/N (%), Escalation, De-escalation, Changes for those not on antibiotics, n/N (%), Continue no antibiotics, Escalation. Row 1: 119/189 (63), 58/189 (30.7), 58/119 (48.7), 49/189 (25.9), 43/189 (22.8), 43/49 (87.8). Row 2: 53/189 (28), 17/189 (9), 17/53 (32.1), 30/189 (15.9), 12/189 (6.3), 12/30 (40). Row 3: 1 (0-2), 0 (0-1), 0 (0-1), 0 (0-0). Row 4: 152/232 (65.5), 61/232 (26.3), 61/152 (40.1), 106/211 (50.2), 53/211 (25.1), 53/106 (50). Row 5: 5/152 (3.3), 6/152 (3.9), 6/5 (100), 1/106 (0.9), 2/106 (1.8), 2/1 (100). Row 6: 147/152 (96.7), 55/152 (36.2), 55/147 (37.4), 105/106 (99.1), 51/106 (48.1), 51/105 (48.6). Row 7: 18/35 (51.4), 23/35 (65.7), 23/18 (100), 26/31 (83.9), 29/31 (93.5), 29/26 (100). Row 8: 17/35 (48.6), 12/35 (34.3), 12/17 (70.6), 5/31 (16.1), 2/31 (6.5), 2/5 (40).

Navigate back to Table 2..

## Table 3. Long description

The table compares antibiotic de-escalation based on BFPP and SOC across various categories. It has 18 rows and 6 columns. Column headers are Theoretical de-escalation, Actual de-escalation, Actual compared to theoretical de-escalation (Based on BFPP), Theoretical de-escalation, Actual de-escalation, Actual compared to theoretical de-escalation (Based on SOC). Row labels include Overall by antibiotic, By antibiotic agent spectrum, By BFPP semi-quantitative level, By antibiotic exposure, By BFPP result time of day, By neutrophil count, By hospital unit, By BFPP guidance comment. Row 1: Overall by antibiotic, n/N (percent), 147/232 (63.4), 55/232 (23.7), 55/147 (37.4), 105/211 (49.8), 51/211 (24.2), 51/105 (48.6). Row 2: By antibiotic agent spectrum, n/N (percent), Gram-positive, 72/147 (49), 31/147 (21.1), 31/72 (43.1), 43/105 (41), 26/105 (24.8), 26/43 (60.5). Row 3: By antibiotic agent spectrum, n/N (percent), Gram-negative, 75/147 (51), 24/147 (16.3), 24/75 (32), 62/105 (59), 24/105 (22.9), 24/62 (38.7). Row 4: By BFPP semi-quantitative level, n/N (percent), Low (10^4-10^5 copies/mL), 43/147 (29.3), 20/147 (13.6), 20/43 (46.5), 29/105 (27.6), 19/105 (18.1), 19/29 (65.5). Row 5: By BFPP semi-quantitative level, n/N (percent), High (10^6-10^7 copies/mL), 41/147 (27.9), 16/147 (10.9), 16/41 (39), 35/105 (33.3), 24/105 (22.9), 24/35 (68.6). Row 6: By antibiotic exposure, n/N (percent), >24 hours, 12/147 (8.2), 4/147 (2.7), 4/12 (33.3), 12/105 (11.4), 7/105 (6.7), 7/12 (58.3). Row 7: By antibiotic exposure, n/N (percent), ≤ 24 hours, 71/147 (48.3), 30/147 (20.4), 30/71 (42.3), 50/105 (47.6), 36/105 (34.3), 36/50 (72). Row 8: By BFPP result time of day, n/N (percent), 0700-1,459, 53/147 (36), 20/147 (13.6), 20/53 (37.7), 39/105 (37.1), 15/105 (14.3), 15/39 (38.5). Row 9: By BFPP result time of day, n/N (percent), 1,500-2,259, 57/147 (38.8), 22/147 (15), 22/57 (38.6), 39/105 (37.1), 16/105 (15.2), 16/39 (41). Row 10: By BFPP result time of day, n/N (percent), 2,300-0659, 37/147 (25.2), 13/147 (8.8), 13/37 (35.1), 27/105 (25.8), 20/105 (19), 20/27 (74.1). Row 11: By neutrophil count, n/N (percent), ANC<1,000 cells/mcL, 23/147 (15.6), 6/147 (4.1), 6/23 (26.1), 19/105 (18.1), 4/105 (3.8), 4/19 (21.1). Row 12: By neutrophil count, n/N (percent), ANC ≥ 1,000 cells/mcL, 124/147 (84.4), 49/147 (33.3), 49/124 (39.5), 86/105 (81.9), 47/105 (44.8), 47/86 (54.7). Row 13: By hospital unit, n/N (percent), Oncology, 84/147 (55.1), 20/147 (13.6), 20/84 (23.8), 63/105 (60), 19/105 (18.1), 19/63 (30.2). Row 14: By hospital unit, n/N (percent), Medicine, 61/147 (41.5), 32/147 (21.7), 32/61 (52.5), 40/105 (38.2), 29/105 (27.6), 29/40 (72.5). Row 15: By BFPP guidance comment, n/N (percent), Negative BFPP prechange, 35/50 (70), 15/50 (30), 15/35 (42.9), 3, 3, 3. Row 16: By BFPP guidance comment, n/N (percent), Negative BFPP postchange, 17/40 (42.5), 9/40 (22.5), 9/17 (52.9), 3, 3, 3.

Navigate back to Table 3..
