# Supplementary material for: Clinical impact of a commercial multiplex pathogen panel for the detection of bacteria in sputum specimens from non-ICU patients with suspected lower respiratory tract infection
Source: Antimicrob Steward Healthc Epidemiol. 2026 Jul 27;6(1):e231. doi: 10.1017/ash.2026.10793 (PMC13419426; doi:10.1017/ash.2026.10793)
Supplement: Gemoules et al. supplementary material [file S2732494X26107931sup001.docx]

**Clinical impact of a commercial multiplex pathogen panel for detection of bacteria in sputum specimens from non-ICU patients with suspected lower respiratory tract infection**

**Supplementary Material**

- **Table S1.** Guidance utilized for appropriateness of antibiotic coverage and actual and theoretical change assessment.

- **Table S2.** BFPP guidance comment changes throughout the study period.
- **Figure S1.** Study design.
- **Table S3**. Distribution of BFPP bacterial target detections by suspected LRTI type.

- **Table S4.** Change in antibiotic spectrum score between specimen collection and BFPP result.

- **Table S5.** Characteristics associated with any change in antibiotic spectrum score.
- **Figure S2.** Antibiotic prescribing.

**Table S1.** Guidance utilized for appropriateness of antibiotic coverage and actual and theoretical change assessment.

| **Adjudication** | **Scenario Description** |
| --- | --- |
| Inappropriate MRSA coverage | Use of an anti-MRSA antibiotic without MRSA identified on BFPP or SOC culture, except for appropriate use of these agents based on antibiotic allergies |
| Inappropriate *P. aeruginosa* coverage | Use of an anti-*P. aeruginosa* antibiotic without *P. aeruginosa* or Enterobacterales identified by BFPP or SOC culture, except for appropriate use of these agents based on antibiotic allergies, for consolidation of therapy (i.e. levofloxacin monotherapy), or in neutropenic patients |
| Appropriate theoretical changes for BFPP results | - Semi-quantitative BFPP level of any titer of a bacterium that does not commonly colonize the respiratory tract:   - Initiate antibiotics, OR   - Broaden antibiotics based on organism, as necessary, OR   - Continue antibiotics if appropriate spectrum - Low semi-quantitative BFPP level (10^4^ copies/mL or 10^5^ copies/mL) of a bacterium that commonly colonizes the respiratory tract:   - Initiate antibiotics, OR   - Continue no antibiotics - BFPP negative:   - Discontinue antibiotics, OR   - Continuation/completion of community acquired LRTI-directed therapy with one of the following options as monotherapy: ceftriaxone, levofloxacin, moxifloxacin, amoxicillin/clavulanate, oral third-generation cephalosporin, oral doxycycline |
| Appropriate theoretical changes for SOC results | - BFPP positive PLUS SOC positive OR upper respiratory flora:   - Continuation/completion of coverage against BFPP positive organism - BFPP positive, SOC negative:   - Discontinue antibiotics, OR   - Continuation/completion of coverage against BFPP positive organism - BFPP negative PLUS SOC negative OR upper respiratory flora result:   - Discontinue antibiotics, OR   - Continuation/completion of community acquired LRTI-directed therapy with one of the following options as monotherapy: ceftriaxone, levofloxacin, moxifloxacin, amoxicillin/clavulanate, oral third-generation cephalosporin, oral doxycycline - BFPP negative, SOC positive:   - Continuation/completion of coverage against SOC positive organism |

Abbreviations: BFPP, BioFire® FilmArray® Pneumonia Panel; LRTI, lower respiratory tract infection; MRSA, methicillin-resistant *Staphylococcus aureus*; SOC, standard of care.

**Table S2.** BFPP guidance comment changes throughout the study period.

| **Timeframe** | **Guidance Comment** |
| --- | --- |
| “Pre-change”  9/1/22-8/31/23 | Rapid molecular analysis has NOT detected bacterial targets (for a list of targets evaluated, refer to the interpretive data for this specimen). Correlation of molecular analysis with culture results is recommended. |
| “Post-change”  9/1/23-8/31/24 | No bacterial targets detected by molecular analysis. A negative molecular panel strongly supports discontinuation of anti-MRSA and anti-pseudomonal therapy for the treatment of pneumonia due to MRSA and *P. aeruginosa* (refer to interpretive data for full list of targets evaluated). Correlation of molecular analysis with culture results is recommended. |

Abbreviations: BFPP, BioFire® FilmArray® Pneumonia Panel.

The above guidance comment statements were reported at the same time as BFPP results.

**Figure S1.** Study design. A total of 189 specimens from 187 adult patients between September 1, 2022, and August 31, 2024 were included.

**
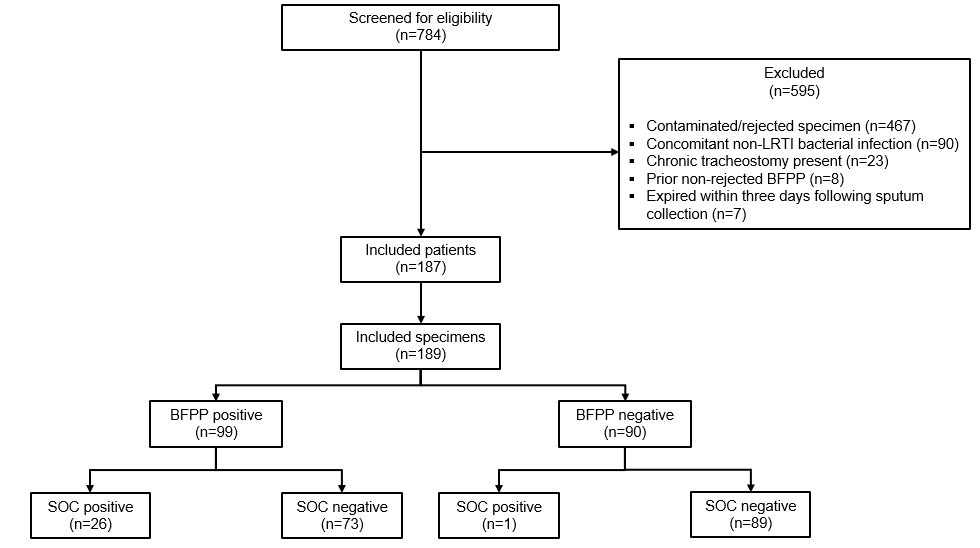
**

Abbreviations: BFPP, BioFire® FilmArray® Pneumonia Panel; LRTI, lower respiratory tract infection; SOC, standard of care.

A single on-panel bacterial target (methicillin-susceptible *Staphylococcus aureus* [MSSA]) was reported as rare growth by SOC culture but not detected by BFPP. Upon review, in these quantities, the MSSA should have been included in upper respiratory flora per routine lab protocol.

Four off-panel bacteria and one off-panel fungus were detected by SOC culture, including *Corynebacterium accolens* (n=1)*, Streptococcus dysgalactiae* (n=1)*, Streptococcus canis* (n=1)*, Stenotrophomonas maltophilia* (n=2)*, and Rhizomucor* (n=1)*.*

**Table S3.** Distribution of BFPP bacterial target detections by suspected LRTI type.

| **Bacterial Target, n (%)^1^** | **Community-Acquired**  **(n=125)** | **Hospital-Acquired**  **(n=64)** | **p-value** | **Total**  **(n=189)** |
| --- | --- | --- | --- | --- |
| *Staphylococcus aureus*  Methicillin-susceptible *S. aureus*  Methicillin-resistant *S. aureus* | 13 (10.4%)  7 (5.6%)  6 (4.8%) | 9 (14.1%)  5 (7.8%)  4 (6.3%) | 0.61  0.75  0.74 | 22 (11.6%)  12 (6.3%)  10 (5.3%) |
| *Streptococcus agalactiae* | 3 (2.4%) | 2 (3.1%) | >0.99 | 5 (2.6%) |
| *Streptococcus pneumoniae* | 19 (15.2%) | 3 (4.7%) | **0.03** | 22 (11.6%) |
| *Streptococcus pyogenes* | 1 (0.8%) | 0 | >0.99 | 1 (0.5%) |
| *Acinetobacter calcoaceticus-baumannii* complex | 0 | 0 | - | 0 |
| *Enterobacter cloacae* complex | 0 | 4 (6.3%) | **0.01** | 4 (2.1%) |
| *Escherichia coli* | 2 (1.8%) | 1 (1.6%) | >0.99 | 3 (1.6%) |
| *Haemophilus influenzae* | 25 (20%) | 13 (20.3%) | >0.99 | 38 (20.1%) |
| *Klebsiella aerogenes* | 0 | 0 | - | 0 |
| *Klebsiella oxytoca* | 0 | 0 | - | 0 |
| *Klebsiella pneumoniae* group | 1 (0.8%) | 2 (3.1%) | 0.26 | 3 (1.6%) |
| *Moraxella catarrhalis* | 10 (8%) | 1 (1.6%) | 0.10 | 11 (5.8%) |
| *Proteus* species | 1 (0.8%) | 0 | >0.99 | 1 (0.5%) |
| *Pseudomonas aeruginosa* | 16 (12.8%) | 9 (14.1%) | 0.99 | 25 (13.2%) |
| *Serratia marcescens* | 4 (3.2%) | 1 (1.6%) | 0.66 | 5 (2.6%) |

Abbreviations: BFPP, BioFire® FilmArray® Pneumonia Panel; LRTI, lower respiratory tract infection.

^1^ Out of 99 positive specimens, 68 had one detection, 23 had two detections, 6 had three detections, 1 had four detections, and 1 had five detections. Co-detections include: *H. influenzae + S. pneumoniae* (n=5), *H. influenzae + S. aureus* (n=3), *P. aeruginosa + S. aureus* (n=3), *K. pneumoniae* group + *S. aureus* (n=1), *Proteus* species + *S. aureus* (n=1), *S. agalactiae + S. aureus* (n=1), *S. pyogenes + S. aureus* (n=1), *H. influenzae + E. coli* (n=1), *H. influenzae + M. catarrhalis* (n=1), *H. influenzae + P. aeruginosa* (n=1), *M. catarrhalis + P. aeruginosa* (n=1), *M. catarrhalis + S. pneumoniae* (n=1), *S. pneumoniae + S. marcescens* (n=1), *P. aeruginosa + E. cloacae* complex (n=1), *P. aeruginosa + E. coli* (n=1), *E. cloacae* complex + *S. agalactiae + S. aureus* (n=1), *M. catarrhalis + S. aureus + S. pneumoniae* (n=1), *H. influenzae + S. agalactiae + S. aureus* (n=1), *M. catarrhalis + S. marcescens + S. pneumoniae* (n=1), *E. coli + K. pneumoniae* group + *S. aureus* (n=1), *M. catarrhalis + P. aeruginosa + S. marcescens* (n=1), *H. influenzae + M. catarrhalis + P. aeruginosa + S. pneumoniae* (n=1), *H. influenzae + M. catarrhalis + P. aeruginosa + S. aureus + S. pneumoniae* (n=1)

**Table S4.** Change in antibiotic spectrum score between sputum collection and BFPP result.

|  | **Median Spectrum Score (IQR)** | | **p-value** |
| --- | --- | --- | --- |
|  | **Sputum Collection** | **BFPP Result** |  |
| All patients^1^ | 27.5 (12.25-40.5) | 27.5 (15.0-40.5) | **0.03** |
| Patients on initial therapy at BFPP collection | 34.0 (27.5-45.0) | 27.5 (19.25-40.5) | **< 0.01** |

Abbreviations: BFPP, BioFire® FilmArray® Pneumonia Panel; IQR, interquartile range.

^1^ The signed rank distribution was significantly different despite the measure of central tendency not being significantly different.

**Table S5.** Characteristics associated with any change in antibiotic spectrum score.

|  | **Odds Ratio**  **(95% CI)** | **p-value** | **Adjusted OR (95% CI)** | **p-value** |
| --- | --- | --- | --- | --- |
| Age ≥ 65 years | 1.85 (1.00-3.45) | 0.05 | - |  |
| Post guidance comment change | 1.15 (0.62-2.11) | 0.65 | - |  |
| Female sex | 0.97 (0.52-1.79) | 0.92 | - |  |
| Race |  |  |  |  |
| White | Ref | Ref |  |  |
| Black | 1.35 (0.70-2.62) | 0.37 | - |  |
| Other | 0.25 (0.01-1.63) | 0.21 |  |  |
| Unable to answer/declined | 2.51 (0.23-54.99) | 0.45 |  |  |
| CCI greater than median of 3 | 0.83 (0.45-1.52) | 0.55 | - |  |
| Neutropenia | 1.05 (0.40-2.77) | 0.90 | - |  |
| Any infection criteria | 1.17 (0.63-2.19) | 0.62 |  |  |
| WBC < 4,000 cells/mcL | 0.77 (0.10-4.77) | 0.78 |  |  |
| WBC > 10,000 cells/mcL | 0.69 (0.37-1.26) | 0.23 | - |  |
| Temperature ≥ 38°C | 1.65 (0.75-3.72) | 0.22 |  |  |
| Temperature ≤ 36°C | 2.38 (0.45-17.50) | 0.32 |  |  |
| Severely immunocompromised^1^ | 1.39 (0.71-2.74) | 0.33 | - |  |
| Nasal cannula respiratory support | 1.07 (0.58-1.96) | 0.83 | - |  |
| Suspected infection type |  |  |  |  |
| Hospital-acquired LRTI | Ref | Ref |  |  |
| **Community-acquired LRTI** | **3.74 (1.88-7.84)** | **<0.01** | **3.50 (1.73-7.39)** | **<0.01** |
| Pre-BFPP antibiotic exposure | 0.63 (0.26-1.47) | 0.29 | - |  |
| Duration antibiotics prior to BFPP | 1.00 (0.99-1.01) | 0.63 | - |  |
| Time to BFPP collection from admission | 0.99 (0.99-1.00) | 0.13 | - |  |
| Active COVID-19 infection | 1.17 (0.27-5.13) | 0.82 | - |  |
| **Spectrum score at BFPP collection (per 10 points)** | **1.23 (1.07-1.46)** | **<0.01** | **1.21 (1.04-1.43)** | **0.02** |

Abbreviations: BFPP, BioFire® FilmArray® Pneumonia Panel; CCI, Charlson comorbidity index; LRTI, lower respiratory tract infection; OR, odds ratio; WBC, white blood cell.

^1^ Severely immunocompromised is defined as patients with a solid organ transplant, hematologic malignancy, hematopoietic cell transplant, HIV or neutropenia (ANC < 1000 cells/mcL).

Variance inflation factor < 3 for all candidate variables for the multivariable model.

*Note.* Community-acquired LRTI was associated with approximately a 3.5-fold higher odds of spectrum change than hospital-acquired LRTI (aOR 3.50, 95% CI 1.73–7.39), consistent with de-escalation of empirical azithromycin (Figure S2). Each 10-point increase in baseline spectrum score conferred 21% higher odds of change (aOR 1.21, 95% CI 1.04-1.43), suggesting broader initial therapy creates greater opportunity for subsequent narrowing.

**Figure S2.** Antibiotic prescribing at specimen collection, post-BFPP result, and post-SOC culture.


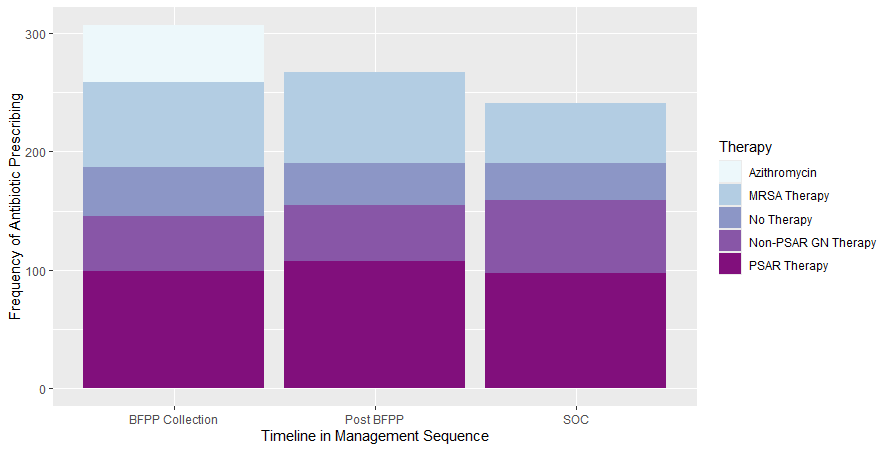


Abbreviations: BFPP, BioFire® FilmArray® Pneumonia Panel; GN, gram-negative; MRSA, methicillin-resistant *Staphylococcus aureus*; PSAR, *Pseudomonas* aeruginosa; SOC, standard of care.
